# The Dark Side of the Sunshine Vitamin: A Case of Acute Renal Failure and Hypercalcaemia From Vitamin D Overconsumption

**DOI:** 10.7759/cureus.70237

**Published:** 2024-09-26

**Authors:** Sheharyar Khan, Muhammad Wasim Tariq, Muhammad Akhtar, Muhammad Tanseer Sibtain Raza, Muhammad Manzoor

**Affiliations:** 1 Emergency Medicine, United Lincolnshire Hospitals National Health Service (NHS) Trust, Grantham and District Hospital, Grantham, GBR; 2 Endocrinology, United Lincolnshire Hospitals National Health Service (NHS) Trust, Pilgrim Hospital, Boston, GBR; 3 Geriatrics, United Lincolnshire Hospitals National Health Service (NHS) Trust, Pilgrim Hospital, Boston, GBR; 4 Critical Care, Nottingham University Hospitals National Health Service (NHS) Trust, Nottingham, GBR; 5 Neurology, University Hospital Southampton National Health Service (NHS) Foundation Trust, Southampton, GBR

**Keywords:** acute kidney injury, case report, hypercalcaemia, vitamin d supplementation, vitamin d toxicity

## Abstract

Vitamin D toxicity is a rare but potentially serious condition that can lead to severe hypercalcaemia and subsequent complications, such as acute renal failure, nephrocalcinosis, congestive heart failure and coronary calcium deposition, resulting in myocardial injury. We present a case of a 76-year-old male patient who developed severe hypercalcaemia and acute kidney injury due to excessive long-term over-the-counter vitamin D supplementation. The patient was initially treated with aggressive intravenous fluids and furosemide. However, a failed effective response prompted the addition of pamidronate, resulting in the gradual improvement of hypercalcaemia and renal function. We have presented, in this case, a rare adverse effect of vitamin D toxicity and its multiorgan effect.

## Introduction

Vitamin D is by far the most commonly prescribed and consumed vitamin and has an important role crucial for calcium homeostasis, bone metabolism, and overall health. While vitamin D deficiency is common, excessive intake leading to toxicity is rare but potentially serious. Vitamin D toxicity can cause severe hypercalcaemia, resulting in complications such as acute kidney injury (AKI), cardiac arrhythmias, and neurological symptoms [1]. In recent years, there has been an increase in reported cases of vitamin D toxicity, likely due to the widespread use of over-the-counter (OTC) supplements and growing awareness of vitamin D's health benefits. In 2021, the America’s Poison Centers (APC) reported 11,718 cases of vitamin D exposure [1]. While the exact incidence remains unclear, these data suggest the growing concern.

Hypercalcaemia in vitamin D toxicity results from increased intestinal calcium absorption and enhanced bone resorption. The excess 25-hydroxyvitamin D bypasses the normal regulatory mechanisms, leading to increased production of 1,25-dihydroxyvitamin D, enhancing intestinal calcium absorption. The resulting hypercalcaemia can lead to AKI through several mechanisms, including renal vasoconstriction, decreased glomerular filtration rate, and calcium-induced nephrogenic diabetes insipidus [2]. Adequate hydration and furosemide are the first-line treatments for hypercalcaemia, followed by bisphosphonate therapy in refractory cases [3].

In this report, we have gone through a case of acute renal failure secondary to hypercalcaemia mediated by excessive serum vitamin D levels.

## Case presentation

A 76-year-old male patient presented to the emergency department (ED) of a local District General Hospital with a six-week history of generalised weakness, dizziness, loss of appetite and weight loss of 5 kg. He also reported increased thirst and excessive urination. His past medical history was significant for hypertension, chronic kidney disease (CKD) stage 3, and presbycusis. He did not take any regular medications but admitted to taking high doses of vitamin D supplements (10,000-20,000 IU daily, increased to 50,000 IU when feeling unwell) for several years without medical supervision. 

On examination, the patient was alert but appeared clinically dehydrated. His observations showed blood pressure of 169/79 mmHg, a regular pulse of 62 beats per minute, a respiratory rate of 16 breaths per minute, a temperature of 36.9°C and oxygen saturation of 95% on room air. Physical examination revealed dry mucous membranes, clear chest sounds and mild right leg tenderness, particularly localised to the ankle, with no signs of injury or infection. There was no evidence of cognitive impairment or focal neurological deficits.

Initial laboratory investigations revealed severe hypercalcaemia (3.33 mmol/L), AKI (creatinine 441 μmol/L), and anaemia (haemoglobin 82 g/L) (Table 1).

**Table 1:** Initial blood investigations of the patient on admission to the emergency department eGFR: estimated glomerular filtration rate; mmol/L: millimoles per litre; μmol/L: micromole per litre; mL/min/1.73 m2: millilitres per minute per 1.73 m2 (of body surface); mg/L: milligrams per litre; g/L: gram per litre; L: litre

Investigation	Reference value	Patient value
Sodium	135-145 mmol/L	142 mmol/L
Potassium	3.5-5.0 mmol/L	3.3 mmol/L
Creatinine	60-110 μmol/L	441 μmol/L
Urea	2.5-7.8 mmol/L	25.4 mmol/L
eGFR	>90 mL/min/1.73 m^2^	10 mL/min/1.73 m^2^
Calcium	2.2-2.6 mmol/L	3.33 mmol/L
C-reactive protein	<5 mg/L	54 mg/L
Haemoglobin	130-180 g/L	83 g/L
White blood cells	4.0-11.0 × 10^9^/L	7.3 × 10^9^/L
Neutrophils	2.0-7.5 × 10^9^/L	5.26 × 10^9^/L

**Figure 1:**
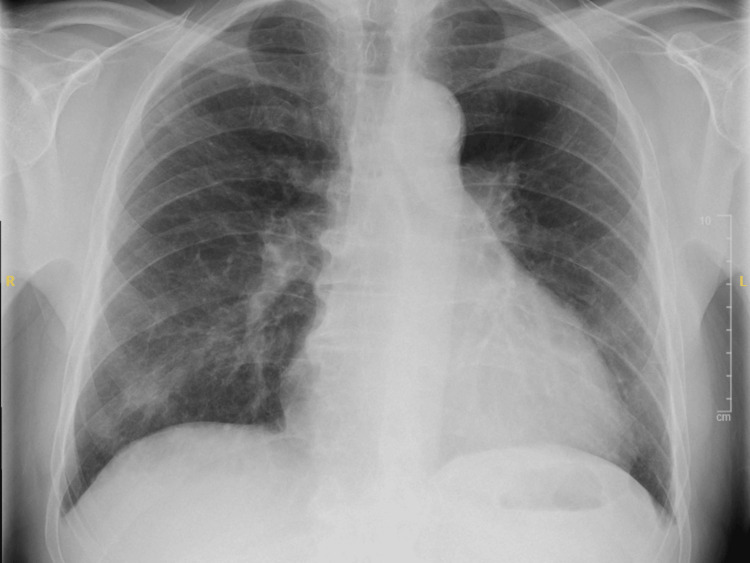
Chest X-ray showing an enlarged heart with a cardiothoracic ratio of 54%. Minimal bilateral pleural effusion and patchy air space opacification in the right lower zone, indicating an underlying infection, can also be seen in this X-ray.

**Figure 2:**
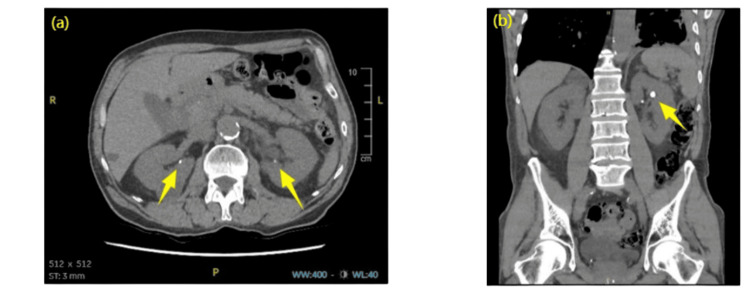
Computed tomography (CT) scan images showing (a) axial section with small non-obstructing calculi visible in the bilateral renal pelvis (yellow arrowhead) and (b) coronal section showing more prominent two small left renal pelvic calculi (yellow arrowhead).

The patient was transferred to the medical admissions unit and was admitted for management of hypercalcaemia. In the workup for his hypercalcaemia, serum 25-hydroxyvitamin D levels were taken, which were markedly elevated at >250 nmol/L (Table 2). Further investigations included normal parathyroid hormone levels, negative serum and urine protein electrophoresis (for myeloma screen) and normal serum ACE levels (to exclude sarcoidosis) (Table 2). Chest X-ray demonstrated bilateral pleural effusions and an increased cardiothoracic ratio of 54% (Figure 1). Apart from significantly elevated vitamin D levels, no other cause of hypercalcaemia was identified, so a plan was made to conduct a computed tomography (CT) to rule out malignancy. The CT scan of the chest, abdomen, and pelvis showed no evidence of malignancy or sarcoidosis but revealed bilateral renal stones, likely indicating nephrocalcinosis (Figure 2).

**Table 2:** Investigations for the causes of hypercalcaemia ACE: angiotensin-converting enzyme; nmol/L: nanomoles per litre; pmol/L: picomoles per litre; U/L: units per litre

Investigation	Reference value	Patient value
25-hydroxyvitamin D	Deficiency: less than 30 nmol/L; insufficiency: 30-50 nmol/L; adequate: greater than 50 nmol/L	>250 nmol/L
Parathyroid hormone	1.6-6.9 pmol/L	2 pmol/L
Serum electrophoresis	-	Unremarkable pattern. No evidence of a monoclonal band.
Urinary protein electrophoresis	-	Unremarkable pattern. No evidence of a monoclonal band.
Serum ACE	20-70 U/L	33 U/L

The management plan for the patient was formulated after multidisciplinary output from renal and endocrine teams. The patient was initially treated with aggressive intravenous fluid resuscitation (0.9% saline at 150 mL/hour) with the cover of furosemide (40 mg twice daily) to prevent fluid overload and to help in the renal excretion of calcium. Despite initial improvement, hypercalcaemia persisted, necessitating the administration of intravenous pamidronate 30 mg on day 21 of admission.

During his hospital stay, the patient also developed type 2 myocardial infarction (MI), with troponin levels peaking at 403 ng/L. A discussion was made with the cardiology team, who suggested a multifactorial cause of the rise in troponin, secondary to ongoing anaemia, acute renal failure, poor left ventricular function and hypercalcaemia. Electrocardiogram (ECG) readings did not show any acute ischaemic changes. The patient required blood transfusions for persistent anaemia (haemoglobin levels fluctuating between 70 g/L to 80 g/L with no obvious cause of bleeding), with a target haemoglobin of >70 g/L.

Over three weeks of his hospital admission, the patient's calcium levels gradually normalised, and renal function improved, though not to baseline levels. The final eGFR at discharge was 25 mL/min/1.73 m² (Table 3).

**Table 3:** Blood investigations after discharge showing improvement in renal functions and normalised serum calcium levels eGFR: estimated glomerular filtration rate

Investigation	Reference value	Patient value
Sodium	135-145 mmol/L	144 mmol/L
Potassium	3.5-5.0 mmol/L	3.7 mmol/L
Creatinine	60-110 μmol/L	213 μmol/L
Urea	2.5-7.8 mmol/L	13.0 mmol/L
eGFR	>90 mL/min/1.73 m^2^	25 mL/min/1.73 m^2^
Calcium	2.2-2.6 mmol/L	2.72 mmol/L

The patient was educated about the risks of excessive vitamin D supplementation and discharged with follow-up appointments for monitoring of renal function and calcium levels.

## Discussion

Vitamin D toxicity, while rare, has become an increasing concern due to the widespread availability of OTC supplements. Our case gives a unique perspective of how excessive and unmonitored consumption of these supplements can result in detrimental and, in some cases, life-threatening complications. The patient's primary symptoms were likely attributable to hypercalcaemia, a direct consequence of elevated vitamin D levels, which resulted in acute renal failure and contributed to the development of cardiovascular complications such as type 2 MI in our case, highlighting the systemic impact of vitamin D toxicity.

Vitamin D toxicity typically occurs with serum 25-hydroxyvitamin D levels exceeding 150 ng/mL (375 nmol/L). In our patient, levels were markedly elevated at >250 nmol/L. The mechanism of toxicity involves increased intestinal calcium absorption and enhanced bone resorption, leading to hypercalcaemia [2]. The patient's prolonged use of high-dose vitamin D supplements (10,000-20,000 IU daily, increased to 50,000 IU when unwell) far exceeded the recommended daily allowance of 600-800 IU for adults [3].

The patient's pre-existing CKD likely predisposed him to more severe complications, as these patients already have altered vitamin D metabolism and are more susceptible to vitamin D toxicity [4]. The AKI observed, in this case, was likely multifactorial, resulting from hypercalcaemia-induced renal vasoconstriction, volume depletion and potential calcium-phosphate crystal deposition in the kidneys, evident from bilateral renal stones [5].

The management of this patient was complex, requiring a multidisciplinary approach. Initial treatment focused on aggressive fluid resuscitation and loop diuretics, which is the first-line approach for hypercalcaemia. The persistent elevation in calcium levels necessitated the use of bisphosphonates (pamidronate), which effectively reduced calcium levels by inhibiting osteoclast activity [6-7].

The gradual normalisation of calcium levels and improvement in renal function over three weeks highlights the extended duration of management in cases of vitamin D toxicity. The residual decrease in eGFR (25 mL/min/1.73 m² at discharge) compared to the patient's baseline emphasises the potential for long-term renal sequelae in such cases, which needs to be closely monitored [8].

This case underlines the growing public health concern surrounding OTC supplement use, particularly in elderly populations. The dramatic increase in vitamin D toxicity cases reported in recent years (1,600% increase from 2005 to 2011 in the United States) reflects the need for improved public education and healthcare provider vigilance regarding appropriate vitamin D supplementation [9].

## Conclusions

Vitamin D toxicity should be considered in patients presenting with unexplained hypercalcaemia, especially those with a history of supplement use. This case has highlighted the importance of judicious use of vitamin D supplements and regular monitoring of calcium and vitamin D levels in patients on long-term supplementation. Healthcare providers should educate patients about the potential risks of OTC supplements and the importance of medical supervision in their use.
